# Trends in Depressive Disorders Among Patients at the Child and Adolescent Psychiatric Unit at Siriraj Hospital Over a 9-Year Period

**DOI:** 10.1192/j.eurpsy.2025.1183

**Published:** 2025-08-26

**Authors:** N. Virikulcharoen, W. Atsariyasing

**Affiliations:** 1 Department of Psychiatry, Mahidol University, Bangkok, Thailand

## Abstract

**Introduction:**

Depression is a significant global health issue among children and adolescents. It has been identified as a leading cause of disability among this population worldwide.

**Objectives:**

This study compares the characteristics and related factors of depressive disorders among child and adolescent patients in Thailand between 2014 and 2022, with the aim of enhancing treatment and screening strategies.

**Methods:**

We included child and adolescent patients diagnosed with depressive disorders at Siriraj Hospital, comprising 93 patients in 2014 and 172 patients in 2022. Data on demographics, personal and social history, diagnoses, comorbidities, and treatment outcomes were collected from medical records. Results were analyzed using relative risk, 95% confidence intervals, and p-values through modified Poisson regression.

**Results:**

The proportion of female patients significantly increased from 49.5% in 2014 to 73.7% in 2022 (p<0.001). The prevalence of domestic violence rose from 7.5% in 2014 to 20.3% in 2022 (p=0.006). Suicidal ideation increased from 16.1% to 51.2% (p<0.001), and self-harming behaviors rose from 22.7% to 57.6% (p<0.001). Referrals to psychologists doubled from 22.6% in 2014 to 43.9% in 2022 (p<0.001), while hospitalization rates within the first year of treatment also increased from 6.5% in 2014 to 15.2% in 2022 (p=0.037). No significant differences were observed in age, family structure, parenting style, disease-triggering factors, psychotic symptoms, comorbidities, medication use, or remission rates between the two years. Our regression analysis indicated that authoritative parenting significantly influenced remission rates, with patients under authoritative parenting being 1.98 times more likely to achieve remission compared to those under other parenting styles (p=0.042).

**Conclusions:**

There is a rising trend in the prevalence of depressive disorders among female child and adolescent patients, accompanied by more severe symptoms such as increased suicidal ideation and self-harming behaviors. Hospitalization rates within the first year of treatment have also increased. Despite these trends, remission rates have remained unchanged. The study highlights the potential role of authoritative parenting in improving remission rates. These findings underscore the need for enhanced screening protocols, updated treatment guidelines, and targeted parental counseling to improve depression management in the future.

**Disclosure of Interest:**

None Declared

